# The survival and cost-effectiveness analysis of adjunctive Chinese medicine therapy for patients with non-small cell lung cancer: a nationwide cohort study in Taiwan

**DOI:** 10.3389/fphar.2024.1378483

**Published:** 2024-06-20

**Authors:** Meng-Bin Tang, Wei-Yin Kuo, Pei-Tseng Kung, Wen-Chen Tsai

**Affiliations:** ^1^ Department of Family Medicine, China Medical University Hospital, Taichung, Taiwan; ^2^ Department of Health Services Administration, China Medical University, Taichung, Taiwan; ^3^ Department of Healthcare Administration, Asia University, Taichung, Taiwan; ^4^ Department of Medical Research, China Medical University Hospital, China Medical University, Taichung, Taiwan

**Keywords:** non-small cell lung cancer, adjunctive Chinese medicine therapy, national health insurance research database, cost-effectiveness analysis, Chinese herbal medicine

## Abstract

**Introduction:** Cancer, particularly lung cancer, is a significant global healthcare challenge. Non-Small Cell Lung Cancer (NSCLC) constitutes 85% of cases. Patients often seek alternative therapies like Chinese medicine alongside Western treatments. This study investigates the survival outcomes and cost-effectiveness of adjunctive Chinese medicine therapy for NSCLC patients in Taiwan.

**Methods:** We utilized the National Health Insurance Research Database in a retrospective cohort study from 2000 to 2018, focusing on NSCLC patients diagnosed between 2007 and 2013. After propensity score matching 1:5 ratio, then compared patients with and without adjunctive Chinese medicine therapy. Survival outcomes, cost-effectiveness, and sensitivity analyses were conducted.

**Results:** The study involved 43,122 NSCLC patients with 5.76% receiving adjunctive Chinese medicine. There is no significant associated between the risk of death and adjuvant Chinese medicine therapy until 181–365 days of adjuvant treatment could reduce the risk of death (HR = 0.88, 95% CI: 0.80–0.98). Cost-effectiveness analysis showed an incremental cost-effectiveness ratio of 880,908 NT$/year.

**Conclusion:** Adjunctive Chinese medicine therapy, particularly when administered for 181–365 days, significantly reduced the mortality risk among stage IV NSCLC patients. The cost-effectiveness aligns with willingness-to-pay thresholds, indicating economic benefit.

## Introduction

The leading cause of death among the Taiwanese population has been cancer for the past 40 years. In 2022, the cancer mortality rate was 222.7 per 100,000 people, with the top three cancer mortality rates being (1) tracheal, bronchus, and lung cancer, (2) liver and intrahepatic bile duct cancer, and (3) colon, rectal, and anal cancer (Statistics on causes of death, 2023). According to a report by the World Health Organization (WHO) in March 2021, cancer was the primary cause of global mortality in 2020, accounting for nearly ten million deaths. The top three cancers of incidence in the same year were breast cancer (2.26 million), lung cancer (2.21 million), and colorectal cancer (1.93 million). The top three cancers causing death were lung cancer (1.80 million), colorectal cancer (935,000), and liver cancer (830,000) (Cancer Organization, 2021). Looking at both the Taiwan and global perspectives, cancer prevention and treatment are significant healthcare concerns, particularly concerning lung cancer. The most common primary malignant tumors in the lungs are categorized as epithelial cancers, broadly divided into small cell lung carcinoma (SCLC) and non-small cell lung carcinoma (NSCLC). NSCLC accounts for approximately 85% of lung cancers and is histologically categorized as adenocarcinoma, squamous cell carcinoma (SCC), and large cell carcinoma (Medscape, 2021). Primary treatment methods include surgical resection, chemotherapy, radiation therapy, targeted therapy, and immunotherapy. Early-stage lung cancer shows promising treatment outcomes, but the mortality rate remains notably high in later stages (Hirsch et al., 2017).

Cancer patients endure significant physical and psychological suffering during the treatment process. The side effects of medications and the possibility of cancer recurrence can greatly impact their outcomes. As a result, cancer patients often seek alternative forms of treatment in addition to conventional Western medicine. These alternatives may include widely practiced therapies like acupuncture and moxibustion, qigong therapy, herbal medicine, and dietary therapy. Some cancer patients embrace Complementary and Alternative Medicine (CAM), and research suggests that they typically use one to two forms of CAM, which can enhance their quality of life (Kuo et al., 2018). In Taiwan, traditional Chinese herbal medications (TCHM) or traditional Chinese medicine (TCM) are accessible to patients through the national health insurance system, leading to 62.5% of the population seeking traditional Chinese medical treatments (Chen et al., 2007). While Western medicine is the predominant global medical approach, TCM plays a vital role as an important form of CAM. It is worth noting that in many Asian countries, TCM remains one of the mainstream medical practices.

In 2017, a study conducted in mainland China focused on the use of TCM as an adjunctive treatment for NSCLC patients. The findings indicated that adjunctive TCM therapy might extend survival and improve the quality of life of NSCLC patients. Additionally, the study suggested that adjunctive TCM therapy could potentially reduce the side effects of radiation and chemotherapy (Liu et al., 2017). In 2018, a study in Taiwan examined the survival analysis of lung cancer patients using herbal medicine as an adjunctive therapy. The study compared 264 patients who used herbal medicine with 528 patients who did not. The results revealed that lung cancer patients using herbal medicine, especially those with high cholesterol and liver cirrhosis, had significantly reduced mortality rates (hazard ratio 0.48, 95% CI: 0.39–0.61, *p* < 0.001) and higher cumulative survival rates (*p* < 0.001) (Li et al., 2018). It is worth noting that previous research on NSCLC and cost-effectiveness mainly focused on screening and Western medicine, with limited literature available regarding the cost-effectiveness of TCM for NSCLC (Klein et al., 2010; Lange et al., 2014; Yang et al., 2017; Kumar et al., 2018).

The purpose of this study is to investigate the differences in the risk of mortality and other related factors among NSCLC patients at different stages based on whether they receive TCM as adjunctive therapy. Additionally, the study aims to explore the variations in healthcare costs and conduct a cost-effectiveness analysis, along with a sensitivity analysis, regarding the use of TCM as adjunctive therapy for NSCLC patients at different stages.

## Materials and methods

### Data sources

This study utilizes a retrospective research methodology and draws data from various sources, including the National Health Insurance research databases (NHIRD) from the Health Data Science Center of the Ministry of Health and Welfare, covering the period from 2000 to 2018. Additionally, the Cancer Registry (2004–2014) from the Health Promotion Administration, the Cause of Death Data provided by the Department of Statistics, and the Household Registration database from the Ministry of the Interior are also used. Taiwan’s National Health Insurance System boasts a remarkable coverage rate of 99.82%, making it a highly representative source for empirical medical data.

### Research participants

The inclusion criteria for the study encompass newly diagnosed NSCLC patients, sourced from the Cancer Registry from 2007 to 2013, with observations tracked until the end of 2018. The identification of lung cancer cases in the Cancer Registry is based on the International Classification of Diseases for Oncology, third edition (ICD-O-3) codes C33∼C34. A total of 76,232 individuals met these criteria. The exclusion criteria were as follows: (1) Patients whose cancer diagnosis date and time are not clear; (2) Patients with small cell lung cancer and ICD-O-3 codes “8,041,” “8,042,” “8,043,” “8,044,” or “8,045”; (3) Patients with *in situ* carcinoma/stage 0 lung cancer; (4) Patients whose cancer stage is unknown; (5) People who have had other cancers before lung cancer; (6) Patients younger than 20 years old; (7) Patients who did not receive active treatment within 1 year; (8) Patients whose primary treatment was not clear. The study included a total of 43,122 individuals after applying these exclusion criteria.

**FIGURE 1:**
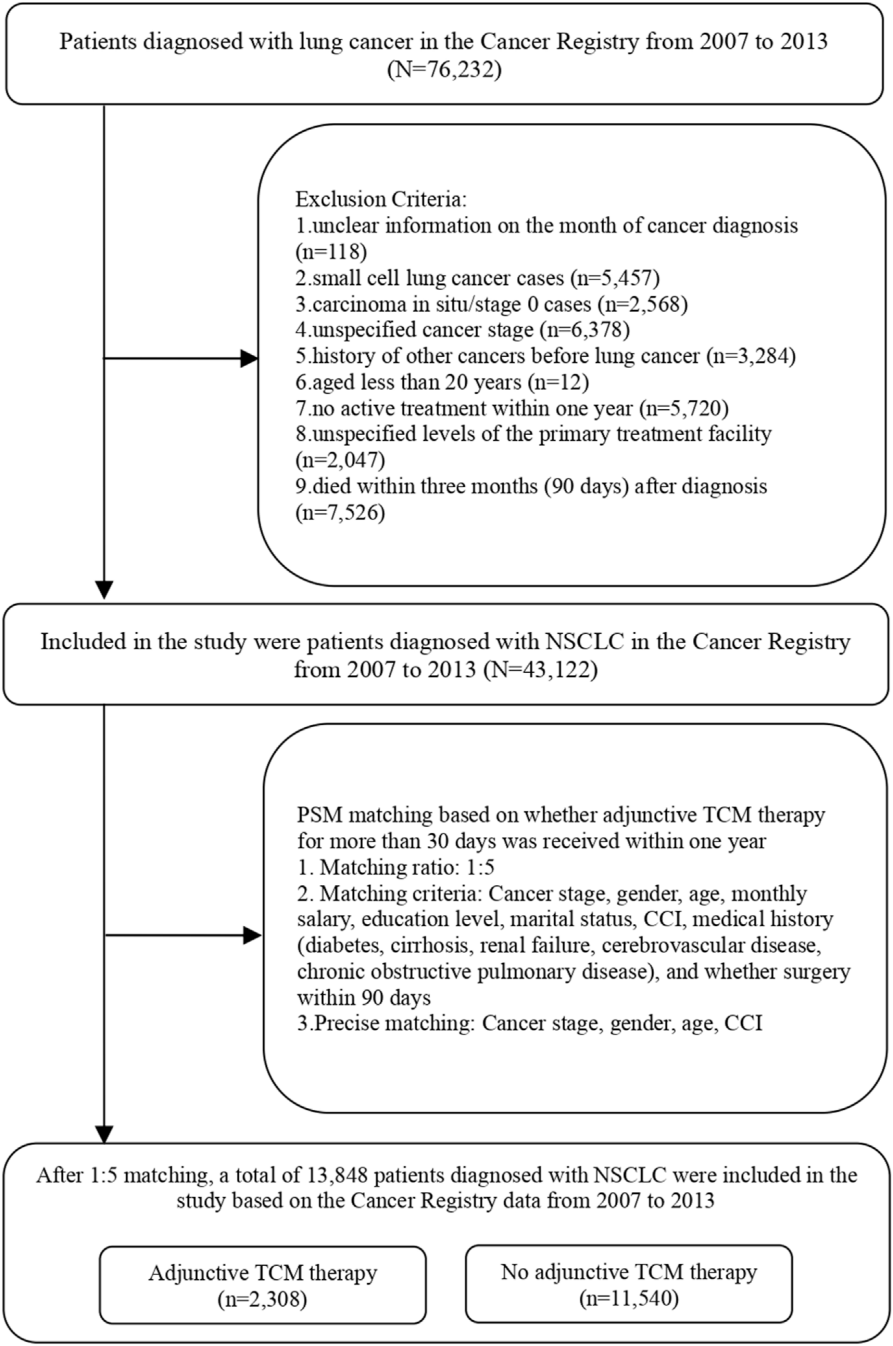
Flow chart.

To mitigate the potential impact of individual characteristics, cancer stage, and disease severity on whether lung cancer patients receive TCM as adjunctive therapy, this study employed the Propensity Score Matching (PSM) method at a 1:5 ratio. PSM attempts to reduce the bias due to confounding variables that could be found in an estimate of the treatment effect obtained from simply comparing outcomes among patients that received the treatment versus those that did not. The purpose was to control for variables including cancer stage, gender, age, salary, education level, marital status, severity of comorbidities, medical history (diabetes, liver cirrhosis, renal failure, cerebrovascular disease, chronic obstructive pulmonary disease), and whether surgery was performed within 90 days. Through precise matching of lung cancer patients who received TCM as adjunctive therapy with those who did not in terms of cancer stage, gender, age, and severity of comorbidities, after matching, there were 2,308 lung cancer patients who received adjunctive TCM therapy and 11,540 who did not. The study included a total of 13,848 patients, as shown in Table 1 and Figure 1.

**TABLE 1:** Descriptive analysis before and after matching whether patients with NSCLC receive adjunctive TCM therapy.

Variable	Pre-matching		Adjunctive TCM therapy					Post-matching		Adjunctive TCM therapy				
	Total		No		Yes		*p*-value	Total		No		Yes		*p*-value
	N	%	N	%	N	%		N	%	N	%	N	%	
Total	43,122	100.00	40,638	94.24	2,484	5.76		13,848	100.00	11,540	83.33	2,308	16.67	
Cancer stage							0.005							1.000
I	8,002	18.56	7,505	93.79	497	6.21		2,838	20.49	2,365	83.33	473	16.67	
II	2,240	5.19	2,081	92.90	159	7.10		690	4.98	575	83.33	115	16.67	
III	9,479	21.98	8,943	94.35	536	5.65		2,940	21.23	2,450	83.33	490	16.67	
IV	23,401	54.27	22,109	94.48	1,292	5.52		7,380	53.29	6,150	83.33	1,230	16.67	
Age							<0.001							1.000
20–54 years/o	8,739	20.27	7,939	90.85	800	9.15		4,470	32.28	3,725	83.33	745	16.67	
55–64 years/o	10,656	24.71	9,859	92.52	797	7.48		4,380	31.63	3,650	83.33	730	16.67	
65–74 years/o	12,042	27.93	11,450	95.08	592	4.92		3,336	24.09	2,780	83.33	556	16.67	
≧75 years/o	11,685	27.10	11,390	97.48	295	2.52		1,662	12.00	1,385	83.33	277	16.67	
Gender							<0.001							1.000
male	24,668	57.21	23,485	95.20	1,183	4.80		6,666	48.14	5,555	83.33	1,111	16.67	
female	18,454	42.79	17,153	92.95	1,301	7.05		7,182	51.86	5,985	83.33	1,197	16.67	
CCI							0.321							1.000
0	41,147	95.42	38,792	94.28	2,355	5.72		13,428	96.97	11,190	83.33	2,238	16.67	
1	1,139	2.64	1,065	93.50	74	6.50		240	1.73	200	83.33	40	16.67	
≥2	836	1.94	781	93.42	55	6.58		180	1.30	150	83.33	30	16.67	
Surgery within 90 days							0.036							0.223
no	6,219	14.42	5,897	94.82	322	5.18		1,733	12.51	1,426	82.29	307	17.71	
yes	36,903	85.58	34,741	94.14	2,162	5.86		12,115	87.49	10,114	83.48	2,001	16.52	
Marital status							<0.001							0.588
Married	30,033	69.65	28,097	93.55	1,936	6.45		11,054	79.82	9,233	83.53	1,821	16.47	
Unmarried	1,919	4.45	1,810	94.32	109	5.68		613	4.43	507	82.71	106	17.29	
Divorced/separated	2,732	6.34	2,592	94.88	140	5.12		730	5.27	597	81.78	133	18.22	
Widowed	6,617	15.34	6,361	96.13	256	3.87		1,451	10.48	1,203	82.91	248	17.09	
missing	1,821	4.22		0.00		0.00								
Education level							<0.001							0.545
Elementary school or below	21,037	48.78	20,216	96.10	821	3.90		4,838	34.94	4,038	83.46	800	16.54	
Junior high school	6,280	14.56	5,901	93.96	379	6.04		2,302	16.62	1,938	84.19	364	15.81	
High school (vocational)	7,704	17.87	7,075	91.84	629	8.16		3,487	25.18	2,898	83.11	589	16.89	
College and above	6,285	14.57	5,673	90.26	612	9.74		3,221	23.26	2,666	82.77	555	17.23	
missing	1,923	4.46		0.00		0.00								
Monthly salary (NT$)							<0.001							0.959
≤17280	11,144	25.84	10,630	95.39	514	4.61		2,944	21.26	2,461	83.59	483	16.41	
17281–22800	17,231	39.96	16,381	95.07	850	4.93		4,840	34.95	4,042	83.51	798	16.49	
22801–36300	6,018	13.96	5,593	92.94	425	7.06		2,418	17.46	2,011	83.17	407	16.83	
36301–57800	5,375	12.46	4,953	92.15	422	7.85		2,275	16.43	1,890	83.08	385	16.92	
≥57801	3,140	7.28	2,888	91.97	252	8.03		1,371	9.90	1,136	82.86	235	17.14	
missing	214	0.50		0.00		0.00								
Comorbidities														
Diabetes	3,548	8.23	3,443	97.04	105	2.96	<0.001	523	3.78	424	81.07	99	18.93	0.175
COPD	2,252	5.22	2,195	97.47	57	2.53	<0.001	276	1.99	223	80.80	53	19.20	0.289
CVD	1,287	2.98	1,239	96.27	48	3.73	0.002	227	1.64	182	80.18	45	19.82	0.231
Liver cirrhosis	563	1.31	540	95.91	23	4.09	0.104	97	0.70	74	76.29	23	23.71	0.083
Renal failure	387	0.90	382	98.71	5	1.29	<0.001	14	0.10	12	85.71	2	14.29	0.550

^a^ Control variables including cancer stage, gender, age, salary, education level, marital status, severity of comorbidities, diabetes, liver cirrhosis, renal failure, cerebrovascular disease, chronic obstructive pulmonary disease and whether surgery was performed within 90 days; precise matching of lung cancer patients who received TCM, as adjunctive therapy with those who did not in terms of cancer stage, gender, age, and severity of comorbidities.

^b^ CCI, charlson comorbidity index; NT$, new taiwan dollar.

### Variable definition and explanation

The patients of this study were followed from the time of voluntary onset confirmation of illness, tracking, and observing until the end of 2018. Non-small cell lung cancer and all other causes of death were defined as causes of death. The date of death and the cause of death were obtained from the Cause of Death Data. The inclusion criteria for the use of TCM were that the first diagnosis code at the time of TCM therapy needed to be lung cancer. Adjunctive TCM therapy within 1 year of diagnosis was defined as not using adjunctive TCM therapy if the duration was less than 30 days and as using adjunctive TCM therapy if the duration was greater than or equal to 30 days (Lin et al., 2015). Furthermore, the use of adjunctive TCM therapy within 1 year of diagnosis was further divided into <30 days, 30–180 days, and 181–365 days for discussion. The experience of using TCM referred to whether patients had consulted the Department of TCM in the 2 years before being diagnosed with NSCLC. The adjunctive TCM therapy included in this study refers specifically to Chinese herbal medicine and does not include other types of TCM therapy.

Basic personal information includes gender, age, marital status, and education level. We categorize gender as either male or female. Age is divided into five groups: 20–54 years, 55–64 years, 65–74 years, and ≥75 years (Huang et al., 2014). Marital status is classified as married, divorced or separated, widowed, or unmarried. The educational level includes elementary school or below, junior high school, high school (vocational), and college and above. Economic status is grouped by monthly salary, from high to low: ≥57801 New Taiwan Dollar (NT$), NT$ 36301–57800, NT$ 22801–36300, NT$ 17281–22800, ≤ NT$ 17280. Environmental factors are differentiated by the degree of urbanization in the residential area. According to Liu and others (2006), Taiwan is divided into seven levels of urbanization, ranging from the highest level (Level 1) to the lowest (Level 7) (Liu et al., 2006). Due to the smaller number of people in Levels 6 and 7, they are combined into Level 5 in this study.

The identification of comorbidities is based on the primary and secondary diagnoses in the medical records of patients in the 2 years before the diagnosis of NSCLC, and the respective disease codes must have occurred two times or more. The specified comorbidities include hypertension (ICD-9 codes 401–405; ICD-10 codes I10-I15), diabetes (ICD-9 code 250; ICD-10 codes E08-E13), hyperlipidemia (ICD-9 code 272; ICD-10 code E78), liver cirrhosis (ICD-9 codes 571; ICD-10 codes K70, K73-K76), renal failure (ICD-9 codes 584–586; ICD-10 codes N17-N19), cerebral vascular disease (ICD-9 codes 430–438; ICD-10 codes I60-I69), chronic obstructive pulmonary disease (ICD-9 codes 491, 492, and 496; ICD-10 codes J41-J44), asthma (ICD-9 code 493; ICD-10 code J45), rheumatoid arthritis (ICD-9 codes 714; ICD-10 codes M05-M06), and chronic mental disorder (ICD-9 codes 295, 296; ICD-10 codes F20, F30-F34, F39). The severity of comorbidities is assessed using the Charlson Comorbidity Index (CCI) defined by Deyo and others (1992) (Deyo et al., 1992). The original CCI disease categories are defined using ICD-9-CM diagnosis or procedure codes, with higher scores indicating more severe comorbidity. CCI scores are calculated by converting the primary and secondary diagnosis codes from the 2 years of medical visits before the patient’s diagnosis, and the cumulative score represents the level of comorbidity.

Hospital attributes refer to the institution where the first treatment (surgery, chemotherapy, radiotherapy, targeted therapy) is administered within 6 months of diagnosis. If there is no treatment, the institution where the main diagnosis first appears is considered the treating institution. Hospital ownership is categorized as public or non-public institutions. Hospital levels include medical centers, regional hospitals, and district hospitals. Cancer staging follows the TNM system (tumor-node-metastasis) and is classified into stages I, II, III, and IV (Kay et al., 2017). Staging information is primarily obtained from the Cancer Registry. Treatment modalities include surgery, chemotherapy, radiotherapy, targeted therapy, surgery combined with other treatments, chemotherapy combined with other treatments, and other treatment combinations. The diagnosis years range from 2007 to 2013.

### Statistical analysis

This study utilized the statistical software SAS 9.4 (SAS Institute Inc., Cary, NC, United States) to analyze and process data, conducting both descriptive and inferential statistics. All statistical analyses used a significance level of *p* < 0.05. Descriptive statistics, including frequencies, percentages, and means, were employed to describe whether NSCLC patients received adjunctive TCM therapy before and after pairing. We performed descriptive analyses on the proportions of NSCLC patients with or without adjunctive TCM therapy across various variables, including demographic, economic, environmental, health-related, cancer staging, hospital attributes, treatment modalities, and diagnosis year.

Inferential statistics include exploring the risk of mortality and its related factors in NSCLC patients with or without adjunctive TCM therapy, economic evaluation, cost-effectiveness analysis (CEA), and sensitivity analysis. The log-rank test was used for bivariate analysis of the mortality outcomes with and without adjunctive TCM therapy. A Cox proportional hazard model was employed to assess the mortality risk during the follow-up period (including overall mortality and mortality due to lung cancer). Censoring was applied for individuals who remained alive until the end of the follow-up period. This study investigated the impact of independent variables and control variables on the survival of NSCLC patients. After controlling for relevant variables, the study explored whether adjunctive TCM therapy is an independent factor affecting patient mortality. Furthermore, the study stratified the analysis by different cancer stages to investigate the 5-year survival differences between those with and without adjunctive TCM therapy.

The economic evaluation in this study is conducted from the perspective of the National Health Insurance Administration (NHIA), with the calculation of healthcare costs primarily based on the NHIRD. The study selected matched research subjects from the NHIRD and tracked all medical records, including outpatient, emergency room, and inpatient visits, for 5 years after the cancer diagnosis. Healthcare costs were calculated for each patient per year, taking into account a discount rate of 3% (Lipscomb, 1996) (Lipscomb, 1996) and adjusting for inflation using the Consumer Price Index (CPI) released by the Directorate-General of Budget, Accounting, and Statistics as the inflation rate for the base year 2013. This study categorizes healthcare costs into three types: medical costs with the primary diagnosis of NSCLC, referred to as primary diagnosis costs; medical costs with either the primary or secondary diagnosis of NSCLC, referred to as primary and secondary diagnosis costs; and the total healthcare costs for all medical visits of NSCLC patients, referred to as total costs, for further analysis.

After matching patients with the same cancer stage into those with and without adjunctive TCM therapy, we conducted a cost-effectiveness analysis using the average person-years of survival and healthcare costs for NSCLC patients within 5 years after diagnosis. This analysis included comparing the medical expenses for each survival year between the group receiving adjunctive TCM therapy and the group without adjunctive TCM therapy. Additionally, we calculated the additional healthcare costs required if the group with adjunctive TCM therapy survived one more year compared to the group without adjunctive TCM therapy. We stratified the study population based on the cancer stage of NSCLC to explore the impact of cancer staging on cost-effectiveness. Key indicators include:

Cost-effectiveness ratio (CER): calculating and comparing the healthcare costs for each survival year between the group with adjunctive TCM therapy and the group without adjunctive TCM therapy.

$$\text{CERy}=\text{Cy}/\text{Ey }\left(\text{NT}\$/\text{year}\right)\text{ CERn}=\text{Cn}/\text{En }\left(\text{NT}\$/\text{year}\right)$$

Cy: Average healthcare costs within 5 years for NSCLC patients with adjunctive TCM therapy.

Cn: Average healthcare costs within 5 years for NSCLC patients without adjunctive TCM therapy.

Ey: Average person-years of survival within 5 years for NSCLC patients with adjunctive TCM therapy.

En: Average person-years of survival within 5 years for NSCLC patients without adjunctive TCM therapy.

CERy: Cost-effectiveness ratio for NSCLC patients with adjunctive TCM therapy.

CERn: Cost-effectiveness ratio for NSCLC patients without adjunctive TCM therapy.

The incremental cost-effectiveness ratio (ICER) represents the additional cost required for one additional year of survival when comparing the group with adjunctive TCM therapy to the group without adjunctive TCM therapy.

$$\text{ICER}=\left(\text{Cy }‐\text{ Cn}\right)\;/\;\left(\text{Ey }‐\text{ En}\right)\;\left(\text{NT}\$/\text{year}\right)$$

To further verify the reliability of the cost-effectiveness of adjunctive TCM therapy for NSCLC, this study utilized bootstrapping simulation. In this simulation test, 1,000 iterations of sampling with replacement were conducted for patients with and without adjunctive TCM therapy. In each iteration, both groups were sampled, with 1,000 and 4,000 individuals, respectively (a total of 5,000 individuals). For each iteration, the incremental cost (△C) and incremental effectiveness (△E) were calculated to create a graph.

## Results

This study included 43,122 patients with NSCLC. Among them, 2,484 patients received adjunctive TCM therapy, accounting for only 5.76%. Looking at cancer stages, the largest number of patients was in stage IV, with 23,401 patients (54.27%). The age distribution showed a higher prevalence in the 65–74 age group (27.93%) and the 75 and above age group (27.10%). There were 24,668 male patients (57.21%) and 18,454 female patients (42.79%). Regarding marital status, the majority were married, accounting for 69.65%, and in terms of education, the highest proportion had an education level of elementary school or below, at 48.78%. Among the patients, 41,147 (95.42%) had a comorbidity severity score of 0, and the most common comorbidity was hypertension, affecting 15.82% of the patients. In terms of treatment methods, most patients (93.16%) had undergone surgical treatment.

A bivariate analysis of the overall cause of death in patients with NSCLC with or without adjunctive TCM therapy (Table 2) revealed that the mortality rate during the follow-up period was 75.17%. The presence or absence of adjunctive TCM therapy was not significantly associated with patient survival or death (*p* > 0.05). Analysis based on the duration of TCM use showed a significant difference in patient survival and death, especially among those using TCM for 181–365 days, effectively reducing the mortality rate to 65.10%. The cancer stage of patients was also significantly associated with survival, with a lower survival rate observed in the later stages of cancer. Table 3 summarizes the analysis of the mortality risk of NSCLC patients with or without adjunctive TCM therapy while controlling for multiple variables (Model 1A). It was found that patients with adjunctive TCM therapy had a significantly reduced risk of mortality compared to those without adjunctive TCM therapy (hazard ratio (HR) = 0.88, 95% confidence interval (CI): 0.83–0.93). Further analysis of the mortality risk based on the duration of adjunctive TCM therapy in NSCLC patients (Model 1B) revealed that patients using adjunctive TCM therapy for 181–365 days had a more effective reduction in the risk of mortality compared to those using adjunctive TCM therapy for less than 30 days (HR = 0.69, 95% CI: 0.63–0.77).

**TABLE 2:** Bivariate analysis of all causes of death in NACLC patients with or without adjuvant TCM therapy.

Variable	Total		Alive		Death		*p*-value
	N	%	N	%	N	%	
Total	13,848	100.00	3,439	24.83	10,409	75.17	
Adjunctive TCM therapy							0.181
no	11,540	83.33	2,840	24.61	8,700	75.39	
yes	2,308	16.67	599	25.95	1,709	74.05	
Days of TCM							<0.001
<30	11,540	83.33	2,840	24.61	8,700	75.39	
30–180	1,692	12.22	384	22.70	1,308	77.30	
181–365	616	4.45	215	34.90	401	65.10	
Cancer stage							<0.001
I	2,838	20.49	2,174	76.60	664	23.40	
II	690	4.98	304	44.06	386	55.94	
III	2,940	21.23	505	17.18	2,435	82.82	
IV	7,380	53.29	456	6.18	6,924	93.82	
Age							<0.001
20–54 years/o	4,470	32.28	1,342	30.02	3,128	69.98	
55–64 years/o	4,380	31.63	1,217	27.79	3,163	72.21	
65–74 years/o	3,336	24.09	700	20.98	2,636	79.02	
≧75 years/o	1,662	12.00	180	10.83	1,482	89.17	
Gender							<0.001
male	6,666	48.14	1,291	19.37	5,375	80.63	
female	7,182	51.86	2,148	29.91	5,034	70.09	
Marital status							<0.001
Unmarried	613	4.43	178	29.04	435	70.96	
Married	11,054	79.82	2,777	25.12	8,277	74.88	
Divorced/separated	730	5.27	224	30.68	506	69.32	
Widowed	1,451	10.48	260	17.92	1,191	82.08	
Education level							<0.001
Elementary school or below	4,838	34.94	871	18.00	3,967	82.00	
Junior high school	2,302	16.62	469	20.37	1,833	79.63	
High school (vocational)	3,487	25.18	965	27.67	2,522	72.33	
College and above	3,221	23.26	1,134	35.21	2,087	64.79	
Monthly salary (NT$)							<0.001
≤17280	2,944	21.26	703	23.88	2,241	76.12	
17281–22800	4,840	34.95	943	19.48	3,897	80.52	
22801–36300	2,418	17.46	639	26.43	1,779	73.57	
36301–57800	2,275	16.43	638	28.04	1,637	71.96	
≥57801	1,371	9.90	516	37.64	855	62.36	
Degree of urbanization							<0.001
1	4,166	30.08	1,220	29.28	2,946	70.72	
2	4,357	31.46	1,081	24.81	3,276	75.19	
3	2,108	15.22	469	22.25	1,639	77.75	
4	1,899	13.71	428	22.54	1,471	77.46	
5	1,318	9.52	241	18.29	1,077	81.71	
CCI							0.053
0	13,428	96.97	3,345	24.91	10,083	75.09	
1	240	1.73	63	26.25	177	73.75	
≥2	180	1.30	31	17.22	149	82.78	
Comorbidities							
hypertension	1,533	11.07	307	20.03	1,226	79.97	<0.001
diabetes	523	3.78	68	13.00	455	87.00	<0.001
hyperlipidemia	238	1.72	59	24.79	179	75.21	1.000
liver cirrhosis	97	0.70	14	14.43	83	85.57	0.024
renal failure	14	0.10	3	21.43	11	78.57	1.000
CVD	227	1.64	22	9.69	205	90.31	<0.001
COPD	276	1.99	25	9.06	251	90.94	<0.001
asthma	178	1.29	28	15.73	150	84.27	0.006
rheumatoid arthritis	33	0.24	11	33.33	22	66.67	0.353
chronic mental disorder	38	0.27	6	15.79	32	84.21	0.270
Hospital ownership							<0.001
non-public	8,381	60.52	1,730	20.64	6,651	79.36	
public	5,467	39.48	1,709	31.26	3,758	68.74	
Hospital level							<0.001
medical centers	9,365	67.63	2,591	27.67	6,774	72.33	
regional hospitals	4,246	30.66	830	19.55	3,416	80.45	
district hospitals	237	1.71	18	7.59	219	92.41	
TCM experience							0.239
No	6,968	50.32	1,700	24.40	5,268	75.60	
Yes	6,880	49.68	1,739	25.28	5,141	74.72	
Treatment modalities							0.423
Surgery	3,383	24.43	1,255	37.10	2,128	62.90	
Chemotherapy	79	0.57	5	6.33	74	93.67	
Radiotherapy	98	0.71	19	19.39	79	80.61	
Targeted therapy	133	0.96	17	12.78	116	87.22	
Surgery + others	9,601	69.33	2,096	21.83	7,505	78.17	
Chemotherapy + others	418	3.02	30	7.18	388	92.82	
Other combinations	136	0.98	17	12.50	119	87.50	
Years of diagnosis							<0.001
2007	1,616	11.67	220	13.61	1,396	86.39	
2008	1,694	12.23	243	14.34	1,451	85.66	
2009	1,925	13.90	405	21.04	1,520	78.96	
2010	1,989	14.36	440	22.12	1,549	77.88	
2011	1,967	14.20	506	25.72	1,461	74.28	
2012	2,182	15.76	736	33.73	1,446	66.27	
2013	2,475	17.87	889	35.92	1,586	64.08	

CCI, charlson comorbidity index; NT$, new taiwan dollar.

**TABLE 3:** Mortality risk and related factors in patients with NSCLC with or without adjunctive TCM therapy (all causes of death).

Variable	Model 1A				Model 1B				Model 2A (survived over 12 months) model 2B							
	HR	95%CI		*p*-value	HR	95%CI		*p*-value	HR	95%CI		*p*-value	HR	95%CI		*p*-value
TCM therapy																
no																
yes	0.88	0.83	0.93	<0.001					1.00	0.95	1.07	0.895				
Days of TCM																
<30																
30–180					0.96	0.90	1.01	0.132					1.06	0.99	1.14	0.097
181–365					0.69	0.63	0.77	<0.001					0.88	0.80	0.98	0.021
Cancer stage																
I																
II	2.78	2.45	3.16	<0.001	2.79	2.46	3.16	<0.001	2.60	2.27	2.98	<0.001	2.60	2.27	2.99	<0.001
III	6.44	5.90	7.03	<0.001	6.40	5.86	6.99	<0.001	5.69	5.18	6.24	<0.001	5.67	5.17	6.23	<0.001
IV	11.50	10.59	12.49	<0.001	11.45	10.55	12.44	<0.001	10.32	9.46	11.26	<0.001	10.31	9.45	11.25	<0.001
Age																
20–54 years/o																
55–64 years/o	1.03	0.97	1.08	0.359	1.02	0.97	1.08	0.399	1.00	0.94	1.07	0.943	1.00	0.94	1.06	0.957
65–74 years/o	1.23	1.16	1.31	<0.001	1.23	1.16	1.31	<0.001	1.17	1.08	1.25	<0.001	1.16	1.08	1.25	<0.001
≧75 years/o	1.84	1.70	1.98	<0.001	1.83	1.70	1.98	<0.001	1.80	1.64	1.97	<0.001	1.79	1.63	1.97	<0.001
Gender																
male																
female	0.68	0.65	0.71	<0.001	0.68	0.65	0.71	<0.001	0.76	0.72	0.79	<0.001	0.76	0.72	0.79	<0.001
Marital status																
Unmarried	1.14	1.03	1.25	0.012	1.13	1.03	1.25	0.014	1.06	0.94	1.20	0.314	1.06	0.94	1.20	0.326
Married																
Divorced/separated	1.08	0.98	1.18	0.122	1.08	0.98	1.18	0.115	1.03	0.93	1.15	0.590	1.03	0.93	1.15	0.582
Widowed	1.04	0.98	1.11	0.229	1.05	0.98	1.12	0.184	1.03	0.95	1.12	0.438	1.04	0.96	1.12	0.388
Education level																
Elementary school or below																
Junior high school	1.00	0.94	1.06	0.960	1.00	0.94	1.06	0.888	1.02	0.95	1.09	0.621	1.02	0.95	1.09	0.640
High school (vocational)	0.91	0.86	0.96	0.001	0.91	0.86	0.96	0.001	0.92	0.86	0.98	0.011	0.92	0.86	0.98	0.011
College and above	0.82	0.76	0.87	<0.001	0.82	0.77	0.87	<0.001	0.84	0.77	0.90	<0.001	0.84	0.78	0.90	<0.001
Monthly salary (NT$)																
≤17280																
17281–22800	0.99	0.93	1.05	0.684	0.99	0.93	1.05	0.671	1.03	0.96	1.11	0.380	1.03	0.96	1.11	0.372
22801–36300	0.95	0.89	1.02	0.126	0.95	0.89	1.02	0.127	0.99	0.91	1.07	0.774	0.99	0.91	1.07	0.787
36301–57800	0.94	0.88	1.01	0.092	0.95	0.89	1.01	0.106	0.98	0.90	1.06	0.552	0.98	0.90	1.06	0.588
≥57801	0.83	0.76	0.90	<0.001	0.83	0.76	0.90	<0.001	0.88	0.80	0.97	0.008	0.88	0.80	0.97	0.008
Degree of urbanization																
1																
2	1.02	0.97	1.07	0.473	1.02	0.97	1.07	0.429	1.06	1.00	1.12	0.065	1.06	1.00	1.12	0.063
3	1.04	0.98	1.10	0.230	1.04	0.98	1.11	0.195	1.04	0.97	1.12	0.252	1.05	0.97	1.13	0.243
4	1.01	0.95	1.08	0.685	1.02	0.96	1.09	0.561	1.02	0.95	1.11	0.564	1.03	0.95	1.11	0.523
5	1.00	0.93	1.08	0.974	1.01	0.93	1.09	0.876	0.98	0.89	1.08	0.698	0.99	0.90	1.08	0.748
CCI																
0																
1	0.88	0.76	1.02	0.091	0.88	0.76	1.02	0.092	0.92	0.78	1.10	0.358	0.93	0.78	1.10	0.370
≥2	1.17	0.99	1.38	0.066	1.17	0.99	1.38	0.058	1.03	0.83	1.28	0.808	1.03	0.83	1.28	0.785
Comorbidities																
hypertension	0.98	0.92	1.05	0.541	0.98	0.92	1.05	0.523	0.99	0.91	1.07	0.742	0.99	0.91	1.07	0.751
diabetes	1.11	1.01	1.23	0.033	1.12	1.01	1.23	0.030	1.07	0.94	1.21	0.327	1.07	0.94	1.21	0.329
hyperlipidemia	0.90	0.77	1.04	0.157	0.89	0.77	1.04	0.148	0.85	0.71	1.02	0.081	0.85	0.71	1.02	0.077
liver cirrhosis	1.50	1.21	1.87	0.000	1.51	1.21	1.88	<0.001	1.46	1.10	1.94	0.008	1.47	1.11	1.95	0.007
renal failure	1.02	0.56	1.86	0.951	1.04	0.57	1.90	0.888	1.18	0.52	2.65	0.699	1.20	0.53	2.70	0.666
CVD	1.30	1.13	1.50	0.000	1.30	1.12	1.50	<0.001	1.31	1.09	1.57	0.004	1.31	1.10	1.58	0.003
COPD	1.41	1.24	1.61	<0.001	1.40	1.23	1.60	<0.001	1.36	1.14	1.61	0.001	1.35	1.13	1.61	0.001
asthma	1.21	1.03	1.43	0.022	1.20	1.02	1.42	0.025	1.28	1.05	1.56	0.015	1.28	1.05	1.56	0.016
rheumatoid arthritis	0.80	0.53	1.22	0.308	0.80	0.52	1.21	0.286	0.90	0.55	1.45	0.650	0.89	0.55	1.43	0.622
chronic mental disorder	1.07	0.75	1.51	0.716	1.07	0.76	1.52	0.695	1.19	0.79	1.79	0.414	1.19	0.79	1.80	0.408
Hospital ownership																
non-public																
public	0.85	0.81	0.89	<0.001	0.85	0.81	0.88	<0.001	0.87	0.82	0.91	<0.001	0.86	0.82	0.91	<0.001
Hospital level																
medical centers																
regional hospitals	1.06	1.02	1.11	0.006	1.06	1.02	1.11	0.008	1.09	1.04	1.15	0.001	1.09	1.04	1.15	0.001
district hospitals	1.29	1.13	1.48	0.000	1.30	1.13	1.49	<0.001	1.32	1.11	1.56	0.002	1.32	1.11	1.57	0.002
TCM experience																
no																
yes	1.00	0.96	1.04	0.835	1.00	0.96	1.04	0.807	1.02	0.97	1.07	0.467	1.02	0.97	1.07	0.478
Treatment modalities																
Surgery	1.45	1.20	1.75	0.000	1.43	1.18	1.73	<0.001	1.13	0.92	1.38	0.246	1.13	0.92	1.38	0.250
Chemotherapy	1.22	0.90	1.64	0.200	1.24	0.92	1.66	0.157	1.18	0.86	1.61	0.311	1.18	0.86	1.62	0.308
Radiotherapy	1.08	0.81	1.45	0.595	1.07	0.80	1.43	0.640	0.92	0.67	1.27	0.613	0.92	0.67	1.26	0.609
Targeted therapy																
Surgery+others	1.41	1.17	1.70	0.000	1.40	1.16	1.68	<0.001	1.11	0.91	1.35	0.300	1.11	0.91	1.35	0.304
Chemotherapy+others	1.21	0.98	1.49	0.083	1.19	0.97	1.47	0.100	1.15	0.92	1.44	0.222	1.15	0.92	1.44	0.223
Other combinations	1.12	0.86	1.44	0.403	1.11	0.86	1.43	0.431	0.97	0.73	1.28	0.817	0.97	0.73	1.28	0.829
Years of diagnosis																
2007																
2008	1.02	0.95	1.10	0.557	1.02	0.95	1.10	0.550	0.98	0.90	1.08	0.711	0.98	0.90	1.07	0.702
2009	0.95	0.88	1.02	0.150	0.95	0.88	1.02	0.185	0.89	0.81	0.97	0.009	0.89	0.82	0.97	0.011
2010	0.89	0.83	0.96	0.002	0.89	0.83	0.96	0.003	0.85	0.78	0.93	<0.001	0.85	0.78	0.93	<0.001
2011	0.81	0.76	0.88	<0.001	0.82	0.76	0.88	<0.001	0.89	0.81	0.97	0.006	0.89	0.82	0.97	0.009
2012	0.75	0.69	0.80	<0.001	0.75	0.70	0.81	<0.001	0.76	0.70	0.84	<0.001	0.77	0.70	0.84	<0.001
2013	0.82	0.76	0.88	<0.001	0.82	0.77	0.89	<0.001	0.73	0.67	0.80	<0.001	0.73	0.67	0.80	<0.001

CCI, Charlson Comorbidity Index; NT$, New Taiwan Dollar; HR, Hazard ratio; CI, confidence interval.

This study also observed an association between patients who survived for more than 12 months and adjunctive TCM therapy. The aim was to eliminate the illusion that patients who survived longer also used TCM for a more extended period, thus excluding the immortal time bias. The findings indicated that, among patients who survived for more than 12 months, the presence or absence of adjunctive TCM therapy was not significantly associated with the risk of mortality (Model 2A). Further analysis of the mortality risk based on the duration of adjunctive TCM therapy in NSCLC patients (Model 2B) revealed that only patients using TCM for 181–365 days had a significant reduction in the risk of mortality compared to those using TCM for less than 30 days (HR = 0.88, 95% CI: 0.80–0.98) (Table 3).

**FIGURE 2:**
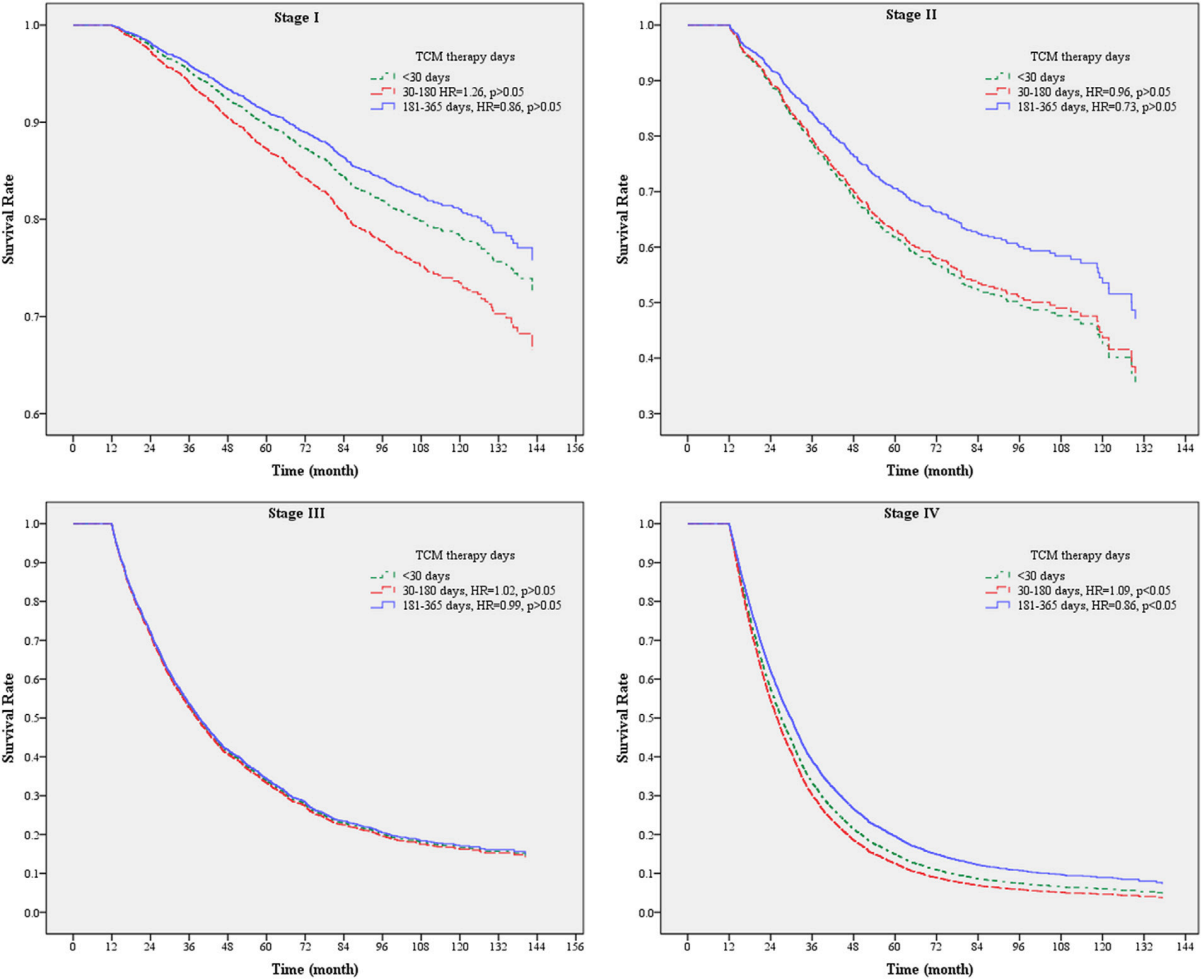
Survival analysis chart of the usage of TCM days in patients with NSCLC of various stages surviving for more than 12 months.

Analyzing the mortality risk of NSCLC patients with or without adjunctive TCM therapy across different stages, the results revealed that the presence or absence of TCM therapy did not show a significant difference in the mortality risk among patients with stages I, II, and III NSCLC (*p* > 0.05). However, for patients in stage IV, adjunctive TCM therapy significantly reduced the risk of mortality compared to those without adjunctive TCM therapy (HR = 0.86, 95% CI: 0.81–0.92). Further analysis of the duration of TCM use and the mortality risk showed that for patients in stages III and IV, using adjunctive TCM therapy for 181–365 days significantly reduced the risk of mortality compared to those using adjunctive TCM therapy for less than 30 days (HR = 0.74, 95% CI: 0.59–0.92; HR = 0.67, 95% CI: 0.59–0.76). Interestingly, for patients in stage I, using adjunctive TCM therapy for 30–180 days did not reduce the risk of mortality compared to those using adjunctive TCM therapy for less than 30 days (HR = 1.29, 95% CI: 1.01–1.64) (Table 4). In this study, the observation of NSCLC patients who survived for more than 12 months revealed that the presence or absence of adjunctive TCM therapy did not show a significant difference in the risk of mortality across different stages (*p* > 0.05). However, for patients in stage IV, using adjunctive TCM therapy for 181–365 days significantly reduced the risk of mortality compared to those using adjunctive TCM therapy for less than 30 days (HR = 0.86, 95% CI: 0.75–0.98) (Figure 2). From these results, it can be concluded that patients in higher stages who use TCM for a longer duration have a greater reduction in the risk of mortality.

**TABLE 4:** Mortality risk and related factors (all causes of death) in patients with NSCLC at various stages with or without adjuvant TCM therapy.

Variable	Model 1A				Model 1B				Model 2A (survived over 12 months) model 2B							
	HR	95%CI		*p*-value	HR	95%CI		*p*-value	HR	95%CI		*p*-value	HR	95%CI		*p*-value
Stage I																
TCM therapy																
no																
yes	1.10	0.89	1.36	0.362					1.11	0.89	1.38	0.345				
Days of TCM																
<30																
30–180					1.29	1.01	1.64	0.042					1.26	0.98	1.63	0.069
181–365					0.81	0.56	1.17	0.250					0.86	0.60	1.25	0.431
Stage II																
TCM therapy																
no																
yes	0.83	0.61	1.12	0.214					0.88	0.63	1.21	0.426				
Days of TCM																
<30																
30–180					0.96	0.68	1.35	0.814					0.96	0.66	1.40	0.838
181–365					0.59	0.34	1.01	0.054					0.73	0.42	1.26	0.251
Stage III																
TCM therapy																
no																
yes	0.90	0.81	1.01	0.071					1.01	0.89	1.14	0.905				
Days of TCM																
<30																
30–180					0.96	0.85	1.08	0.499					1.02	0.88	1.17	0.833
181–365					0.74	0.59	0.92	0.007					0.99	0.79	1.24	0.904
Stage IV																
TCM therapy																
no																
yes	0.86	0.81	0.92	<0.001					1.01	0.94	1.09	0.714				
Days of TCM																
<30																
30–180					0.95	0.88	1.02	0.124					1.09	1.00	1.19	0.044
181–365					0.67	0.59	0.76	<0.001					0.86	0.75	0.98	0.022

^a^ Other relevant variables have been controlled; HR, hazard ratio; CI, confidence interval.

The analysis of mortality risk in the previous results was based on the overall causes of death among patients. We further conducted an analysis, specifically considering deaths due to lung cancer (Table 5). The findings showed that the presence or absence of adjunctive TCM therapy did not significantly affect the mortality risk for patients who survived for more than 12 months (*p* > 0.05) (Model 2A). However, among those using adjunctive TCM therapy for 181–365 days, there was a significant reduction in mortality risk compared to those using it for less than 30 days (HR = 0.90, 95% CI: 0.80–1.00) (Model 2B). Further analysis was carried out for different stages of lung cancer, indicating that the use of TCM did not affect the mortality risk across various stages, except for stage IV patients. In stage IV, using adjunctive TCM therapy for 181–365 days significantly lowered the mortality risk compared to using it for less than 30 days (HR = 0.86, 95% CI: 0.76–0.99). These results demonstrate that the analysis of mortality risk, considering overall causes and causes specifically related to lung cancer, yielded nearly consistent results.

**TABLE 5:** Mortality risk from lung cancer and related factors in NSCLC patients with or without adjunctive TCM therapy.

Variable	Model 1A				model 1B				Model 2A (survived over 12 months) model 2B							
	HR	95%CI		*p*-value	HR	95%CI		*p*-value	HR	95%CI		*p*-value	HR	95%CI		*p*-value
No staging																
TCM therapy																
no																
yes	0.88	0.84	0.93	<0.001					1.01	0.95	1.07	0.861				
Days of TCM																
<30																
30–180					0.96	0.90	1.02	0.147					1.06	0.98	1.14	0.132
181–365					0.71	0.64	0.78	<0.001					0.90	0.80	1.00	0.047
Stage I																
TCM therapy																
no																
yes	1.09	0.85	1.39	0.496					1.09	0.85	1.40	0.505				
Days of TCM																
<30																
30–180					1.31	0.99	1.73	0.062					1.27	0.95	1.70	0.109
181–365					0.74	0.47	1.16	0.184					0.80	0.51	1.25	0.318
Stage II																
TCM therapy																
no																
yes	0.88	0.64	1.21	0.425					0.94	0.67	1.33	0.723				
Days of TCM																
<30																
30–180					0.98	0.68	1.41	0.910					0.97	0.65	1.46	0.890
181–365					0.70	0.41	1.21	0.206					0.88	0.51	1.54	0.656
Stage III																
TCM therapy																
no																
yes	0.90	0.80	1.01	0.074					1.01	0.88	1.15	0.924				
Days of TCM																
<30																
30–180					0.95	0.84	1.08	0.415					1.00	0.86	1.16	1.000
181–365					0.77	0.61	0.96	0.020					1.02	0.81	1.29	0.841
Stage IV																
TCM therapy																
no																
yes	0.87	0.81	0.93	<0.001					1.01	0.94	1.09	0.736				
Days of TCM																
<30																
30–180					0.95	0.88	1.02	0.168					1.09	1.00	1.19	0.057
181–365					0.68	0.60	0.77	<0.001					0.86	0.76	0.99	0.029

^a^ Other relevant variables have been controlled; HR, hazard ratio; CI, confidence interval.

Whether NSCLC patients used adjunctive TCM therapy was analyzed for healthcare costs over a 5-year period. The costs were categorized into medical expenses with the primary diagnosis of NSCLC, medical expenses with either the primary or secondary diagnosis of NSCLC, and the total healthcare expenses for all medical visits. Observing the 5-year 3% discounted and CPI-adjusted average annual costs per person for the primary diagnosis, it was NT$ 975,557. For those without adjunctive TCM therapy, the cost was NT$957,598, while for those with adjunctive TCM therapy, it was NT$1,065,355. The average annual costs per person for primary and secondary diagnoses were NT$ 1,221,444. For those without adjunctive TCM therapy, the cost was NT$ 1,198,137, and for those with adjunctive TCM therapy, it was NT$ 1,337,979. The average annual total cost per person was NT$1,261,660. For those without adjunctive TCM therapy, the cost was NT$ 1,236,988, and for those with adjunctive TCM therapy, it was NT$ 1,385,021. From these results, it can be observed that the costs for patients with adjunctive TCM therapy are higher than those without adjunctive TCM therapy. Total costs and primary and secondary diagnosis costs are relatively close, but there is a larger difference in costs for the primary diagnosis (Table 6).

**TABLE 6:** Cost-effectiveness of 5-year used adjunctive TCM therapy for patients with NSCLC.

	Five years 3% discount and CPI healthcare costs		
	Total	No TCM	TCM
Primary diagnosis costs			
Average costs (C)	975,557	957,598	1,065,355
Average survival (E)	2.791	2.763	2.931
CER (C/E)	349,597	346,640	363,533
ICER (△C/△E)	641,236		
Primary and secondary diagnosis costs			
Average costs (C)	1,221,444	1,198,137	1,337,979
Average survival (E)	3.564	2.763	2.931
CER (C/E)	342,728	433,713	456,561
ICER (△C/△E)	832,163		
Total costs			
Average costs (C)	1,261,660	1,236,988	1,385,021
Average survival (E)	3.564	2.763	2.931
CER (C/E)	354,012	447,777	472,614
ICER (△C/△E)	880,908		

C, average healthcare costs per patient; E, Average person-years of survival; CER, Cost-effectiveness ratio; ICER, Incremental cost-effectiveness ratio; CPI, consumer price index.

This study further analyzed the medical expenses for different stages of cancer, including primary diagnosis costs, primary and secondary diagnosis costs, and total costs. Observing the 5-year 3% discounted and CPI-adjusted average annual medical expenses per person, the highest expenses were observed for cancer stage III (total costs with or without adjunctive TCM therapy were NT$ 1,544,137 and NT$ 1,395,542, respectively). Following that, in descending order, were stage IV (total costs with or without adjunctive TCM therapy were NT$ 1,497,446 and NT$ 1,338,481, respectively), stage II (total costs with or without adjunctive TCM therapy were NT$ 1,364,661 and NT$ 1,239,668, respectively), and stage I (total costs with or without adjunctive TCM therapy were NT$ 932,785 and NT$ 808,156, respectively) (Table 7).

**TABLE 7:** Cost-effectiveness of 5-year adjunctive TCM therapy for patients with NSCLC at various stages.

	Five years 3% discount and CPI healthcare costs																
	Primary diagnosis costs					Primary and secondary diagnosis costs						Total costs					
	Total	No TCM		TCM		Total		No TCM		TCM		Total		No TCM		TCM	
stage I																	
Average costs (C)	616,975		599,103		706,333	771,805	753,518				863,241	828,928			808,156		932,785
Average survival (E)	4.679		4.688		4.637	4.679	4.688				4.637	4.679			4.688		4.637
CER (C/E)	131,847		127,793		152,340	164,934	160,731				186,181	177,141			172,386		201,180
ICER (△C/△E)	−2,081,823					−2,130,237							−2,419,623				
stage II																	
Average costs (C)	987,827		981,412		1,019,900	1,208,708			1,187,956		1,312,463		1,260,500		1,239,668		1,364,661
Average survival (E)	3.751		3.730		3.856	3.751			3.730		3.856		3.751		3.730		3.856
CER (C/E)	263,360		263,119		264,526	322,248			318,494		340,406		336,056		332,357		353,944
ICER (△C/△E)	306,297					990,850							994,722				
stage III																	
Average costs (C)	1,116,834		1,098,103		1,210,491	1,378,993			1,354,579		1,501,065		1,420,308		1,395,542		1,544,137
Average survival (E)	2.673		2.629		2.890	2.673			2.629		2.890		2.673		2.629		2.890
CER (C/E)	417,872		417,667		418,805	515,961			515,218		519,338		531,419		530,799		534,240
ICER (△C/△E)	430,261					560,799							568,869				
stage IV																	
Average costs (C)	1,056,023		1,037,257		1,149,849	1,332,781			1,307,746		1,457,957		1,364,976		1,338,481		1,497,446
Average survival (E)	2.021		1.985		2.204	2.021			1.985		2.204		2.021		1.985		2.204
CER (C/E)	522,455		522,623		521,701	659,378			658,908		661,493		675,306		674,395		679,410
ICER (△C/△E)	513,356					684,880							724,791				

C, average healthcare costs per patient; E, Average person-years of survival; CER, Cost-effectiveness ratio; ICER, Incremental cost-effectiveness ratio; CPI, consumer price index.

The cost-effectiveness analysis of NSCLC patients with or without adjunctive TCM therapy, using total costs as an example, revealed a CER of 472,614 NT$/year for the group with adjunctive TCM therapy and 447,777 NT$/year for the group without adjunctive TCM therapy. The ICER, representing the additional medical expenses needed for an extra year of survival in the group with adjunctive TCM therapy compared to the group without, was 880,908 NT$/year (Table 6). However, for the ICER in primary and secondary diagnosis costs, it was 832,163 NT$/year, and for primary diagnosis costs, it was 641,236 NT$/year. Different stages of NSCLC showed varying CERs (Table 7). Whether it was total costs, primary and secondary diagnosis costs, or primary diagnosis costs, the CER increased with the cancer stage. However, the ICER did not necessarily increase with the cancer stage.

Taking total costs as an example, the CER for NSCLC patients with adjunctive TCM therapy, from stage I to stage IV, is as follows: 201,180 NT$/year, 353,944 NT$/year, 534,240 NT$/year, and 679,410 NT$/year, respectively. For NSCLC patients without adjunctive TCM therapy, the CER from stage I to stage IV is as follows: 172,386 NT$/year, 332,357 NT$/year, 530,799 NT$/year, and 674,395 NT$/year, respectively. The ICER does not follow a direct proportion to the cancer stage. For stage I patients, the ICER is negative because the group with adjunctive TCM therapy, on average, has a shorter survival. Among the stages, stage II patients have the highest ICER (994,722 NT$/year), stage III patients have the lowest ICER (568,869 NT$/year), and stage IV patients have an intermediate ICER (724,791 NT$/year) (Table 7).

The analysis of medical expenses focusing on primary and secondary diagnoses reveals a pattern similar to the total costs. The order of ICER among patients at different stages aligns with the total costs. The sequence of ICER from highest to lowest is stage II patients (990,850 NT$/year), stage IV patients (684,880 NT$/year), and stage III patients (560,799 NT$/year). However, when considering medical expenses for the primary diagnosis, the ICER is directly proportional to the cancer stage. The sequence of ICER from highest to lowest is stage IV patients (513,356 NT$/year), stage III patients (430,261 NT$/year), and stage II patients (306,297 NT$/year) (Table 7).

To assess whether a healthcare intervention is cost-effective, researchers often use a cost-effectiveness threshold. Previous studies commonly utilized the willingness-to-pay (WTP) amount as an indicator. The WHO recommends using three times the gross domestic product (GDP) as the threshold (World Health Organization, 2009; Robinson et al., 2017). Many of Taiwan’s studies also follow the WHO’s suggestion of using three times the GDP to determine the cost-effectiveness standard. Therefore, this study adopts the average *per capita* GDP from 2007 to 2013, which is NT$ 597,354, and calculates 3 times the GDP, which equals NT$ 1,792,062, as the threshold for measurement. Overall, for NSCLC patients using adjunctive TCM therapy, the ICER for total healthcare expenses is 880,908 NT$/year, which is less than NT$ 1,792,062, indicating that using adjunctive TCM therapy is cost-effective.

**FIGURE 3:**
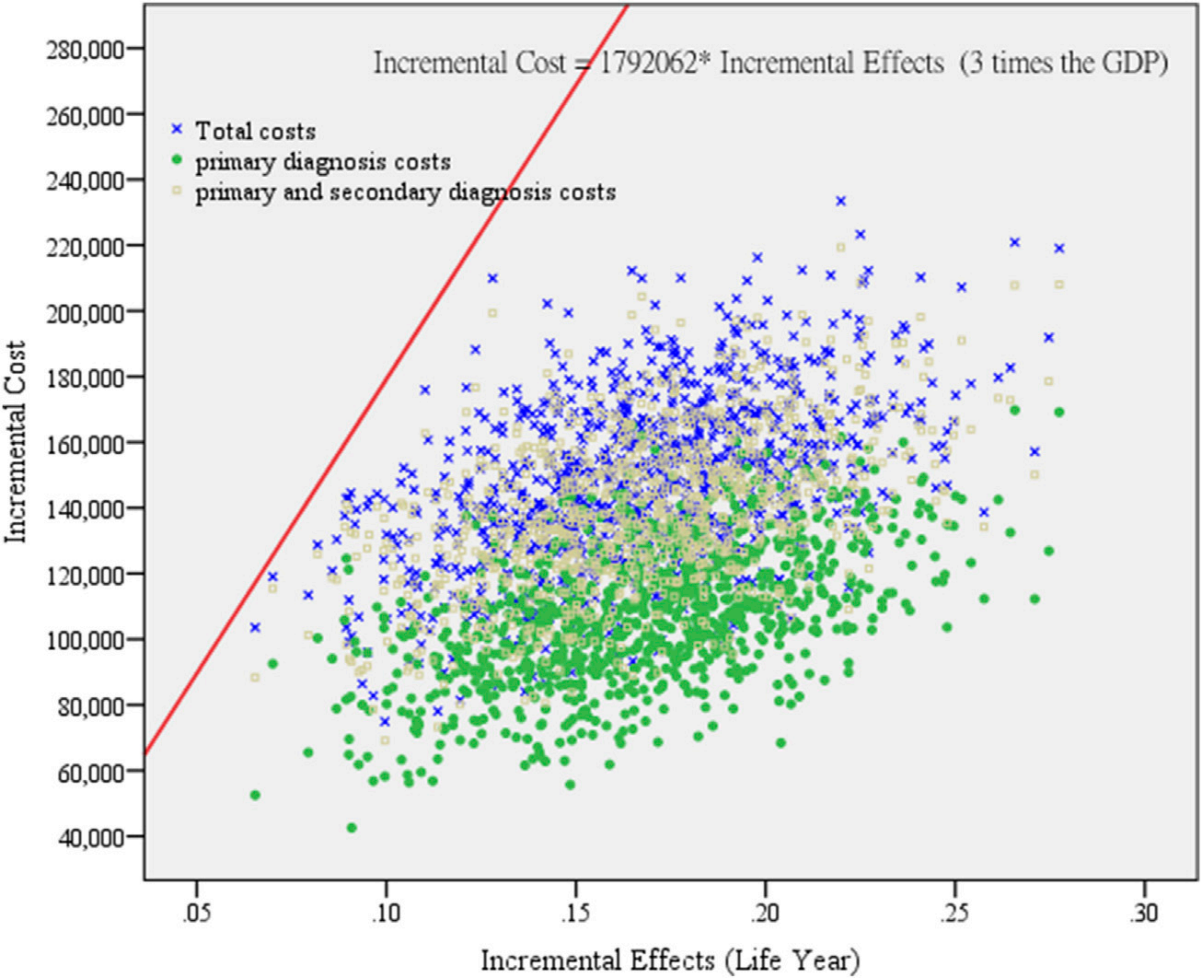
Cost-effectiveness reliability testing of repeated sampling among lung cancer patients. (According to primary diagnosis, primary and secondary diagnosis, and total cost distribution.). This study self-sampled 1,000 groups of NSCLC patients, and compared to those who did not receive adjunctive TCM therapy, the cost-effectiveness of patients receiving adjunctive TCM therapy falls within an acceptable range (within 3 times GDP) for primary diagnosis costs, primary and secondary diagnosis costs, and total costs.

Furthermore, to assess the reliability of cost-effectiveness, a bootstrapping simulation test was conducted for NSCLC patients, with repeated sampling distinguishing costs by primary diagnosis, primary and secondary diagnosis, and total costs (Figure 3). Random sampling with replacement was performed 1,000 times, with each group consisting of NSCLC patients matched in a 1:4 ratio (1,000 patients with and 4,000 patients without adjunctive TCM therapy, totaling 5,000 patients). The analysis results showed that 100% of the simulations fell within the cost-effectiveness acceptability curves, i.e., within 3 times the GDP. The bootstrapping simulation results indicate that the cost-effectiveness of this study aligns well with the economic benefits of the country. Regardless of distinguishing costs by primary diagnosis, primary and secondary diagnosis, or total costs, the data points for each group are highly concentrated, demonstrating the robustness of the cost-effectiveness analysis results.

This study focuses on adjunctive TCM therapy, with an emphasis on Chinese herbal medicine. Table 8 presents the 20 most commonly used single herbs and formulas in adjunctive therapy for NSCLC patients. There are 439 single herbal medicine items, totaling 440,971 prescription records, and 456 formula items, totaling 251,750 prescription records. The ten most frequently used single herbs are, in order: Scleromitrion diffusum, Platycodon grandiflorus, Astragalus mongholicus, Fritillaria thunbergii, Houttuynia cordata, Scutellaria barbata, Scutellaria baicalensis, Prunus mandshurica, Rheum rhabarbarum, and Taraxacum mongolicum. The ten most frequently used formulas are, in order: San Jhong Kuei Jian Tang, Siang Sha Liou Jyun Zih Tang, Xiao Chai Hu Tang, Jiawei Siaoyao San, Bai He Gu Jin Tang, Qing Zao Jiu Fei Tang, Bansia Siexin Tang, Bu Zhong Yi Qi Tang, Ganlu Yin, and Wen Dan Tang. Scleromitrion diffusum (4.23%) has significantly higher usage than other single herbs. The most frequently used formula is San Jhong Kuei Jian Tang (1.73%), slightly higher than the second most used formula, Siang Sha Liou Jyun Zih Tang (1.41%).

**TABLE 8:** The most common TCM herbs and formulas used by NSCLC patients.

Order	Herb	N	%	Order	Formula	N	%
1	Scleromitrion diffusum (Willd.) R.J.Wang [Rubiaceae]	18,652	4.23	1	San Jhong Kuei Jian Tang	7,622	1.73
2	Platycodon grandiflorus (Jacq.) A.DC. [Campanulaceae]	8,965	2.03	2	Siang Sha Liou Jyun Zih Tang	6,239	1.41
3	Astragalus mongholicus Bunge [Faboideae]	8,340	1.89	3	Xiao Chai Hu Tang	5,758	1.31
4	Fritillaria thunbergii Miq. [Liliaceae]	8,333	1.89	4	Jiawei Siaoyao San	5,742	1.30
5	Houttuynia cordata Thunb. [Saururaceae]	7,950	1.80	5	Bai He Gu Jin Tang	5,494	1.25
6	Scutellaria barbata D.Don [Lamiaceae]	7,907	1.79	6	Qing Zao Jiu Fei Tang	5,329	1.21
7	Scutellaria baicalensis Georgi [Lamiaceae]	6,898	1.56	7	Bansia Siexin Tang	5,306	1.20
8	Prunus mandshurica (Maxim.) Koehne [Rosaceae]	6,692	1.52	8	Bu Zhong Yi Qi Tang	4,488	1.02
9	Rheum rhabarbarum L. [Polygonaceae]	6,183	1.40	9	Ganlu Yin	4,278	0.97
10	Taraxacum mongolicum Hand.-Mazz. [Crepidinae]	6,148	1.39	10	Wen Dan Tang	4,233	0.96
11	Magnolia officinalis Rehder and E.H.Wilson [Magnoliaceae]	5,826	1.32	11	Mai Men Dong Tang	3,959	0.90
12	Salvia miltiorrhiza Bunge [Lamiaceae]	5,813	1.32	12	Xue Fu Zhu Yu Tang	3,872	0.88
13	Atractylodes macrocephala Koidz. [Asteraceae]	5,694	1.29	13	Ping Wei San	3,748	0.85
14	Wolfiporia cocos (Schw.) Ryv. and Cilbn. [Polyporaceae]	5,533	1.25	14	Zhi Gan Cao Tang	3,748	0.85
15	Pinellia ternata (Thunb.) Makino [Araceae]	5,141	1.17	15	Sha Shen Mai Dong Tang	3,621	0.82
16	Liriope longipedicellata F.T.Wang and Tang [Asparagaceae]	5,001	1.13	16	Ma Xing Gan Shi Tang	3,276	0.74
17	Corydalis yanhusuo (Y.H.Chou and Chun C.Hsu) W.T.Wang ex Z.Y.Su and C.Y.Wu [Papaveraceae]	4,955	1.12	17	Sheng Mai Yin	3,220	0.73
18	Descurainia sophia (L.) Webb ex Prantl [Brassicaceae]	4,604	1.04	18	Ding Chuan Tang	3,209	0.73
19	Glycyrrhiza uralensis Fisch. ex DC. [Faboideae]	4,211	0.95	19	Xin Yi Qing Fei Tang	3,086	0.70
20	Codonopsis pilosula (Franch.) Nannf. [Campanulaceae]	3,986	0.90	20	Xiao Qing Long Tang	2,980	0.68

## Discussion

The results of this study found that, without excluding immortal time bias, there is an association between adjunctive TCM therapy and the survival of patients with NSCLC. Those who received adjunctive TCM therapy had a 12% lower risk of mortality, and those using adjunctive TCM therapy for 181–365 days had a 31% lower risk of mortality. Previous studies have also shown a 35% reduction in the risk of mortality for those receiving adjunctive TCM therapy (Yeh et al., 2020) and a 71% reduction in the risk of mortality for those using adjunctive TCM therapy for 181–365 days (Xu et al., 2021). Taiwan published literature in 2020 (Yeh et al., 2020) based on 1871 lung cancer patients from two teaching hospitals in Taiwan from 2003 to 2016. The analysis was conducted without matching to determine the results of adjunctive TCM therapy. The research published in mainland China in 2021 (Xu et al., 2021) analyzed the results of adjunctive TCM therapy for 136 small cell lung cancer patients in a teaching hospital in mainland China from 2015 to 2018. From the above, it can be seen that the previous two studies concluded a greater reduction in the risk of mortality with adjunctive TCM therapy than this study. The reasons may include limitations in patient sources to one to two hospitals, a smaller number of lung cancer patients, less rigorous study designs (e.g., lack of matching), the inclusion of all types of lung cancer, or a focus only on small cell lung cancer, which is different from this study. Additionally, previous studies were conducted by specialized Chinese and Western medicine teams in teaching hospitals dedicated to lung cancer treatment, which differs from the medical records of lung cancer patients in the NHIRD. A specialized lung cancer treatment team may achieve better treatment outcomes.

To avoid immortality bias in the analysis of the survival of patients with NSCLC receiving adjunctive TCM therapy, the focus was on exploring patients who survived for more than 12 months. The analysis revealed that there was no significant association between adjunctive TCM therapy and the survival of patients with NSCLC. Further analysis of the duration of Chinese medicine usage showed that patients using adjunctive TCM therapy for 181–365 days could significantly reduce the risk of mortality by 12% (HR = 0.88, 95% CI: 0.80–0.98), and stage IV patients could reduce the risk by 14% (HR = 0.86, 95% CI: 0.81–0.92). When discussing adjunctive TCM therapy with clinical practitioners and patients with NSCLC, it is crucial to clearly inform patients that the duration of Chinese medicine usage should be sufficiently long to achieve significant efficacy. There is no apparent benefit to taking it for less than 6 months. From a survival perspective, encouraging stage IV NSCLC patients to use adjunctive TCM therapy for 181 days or more can increase the average 5-year survival rate from 10.69% to 17.53%. Similar results were reported in studies from mainland China in 2021 and Taiwan in 2020. These studies found that adjunctive TCM therapy for more than 6 months (or 180 days) could extend survival, with significant differences observed in stage IV patients for various cancer types (Yeh et al., 2020; Xu et al., 2021). However, this study had a larger number of patients, a longer follow-up time, and addressed immortal time bias more comprehensively.

In this study, the analysis of mortality risk compared overall mortality with mortality specifically due to lung cancer, and the results were similar. It can be inferred that the majority of deaths in patients with NSCLC are attributed to lung cancer. The 5-year survival rate for patients is only 31.55%. In addition to other relevant factors, the study found that higher cancer stages, older age, male gender, lower education level, comorbidities such as liver cirrhosis, cerebrovascular disease, chronic obstructive pulmonary disease, asthma, non-public hospital patients, and lower hospital levels were associated with higher mortality risks. These findings align with some aspects of previous studies (Liao et al., 2017; Yeh et al., 2020; Xu et al., 2021). However, in contrast to prior research, this study did not find an increased risk of mortality for patients with comorbid diabetes or renal failure.

The existing literature has not directly addressed the CEA of adjunctive TCM therapy for NSCLC patients. There is some relevant literature, such as a study from mainland China in 2023 that reported on medical expenses for lung cancer inpatients. Between 2010 and 2016, there were a total of 47,393 lung cancer inpatients, and the median annual total medical expenses (Western medicine + Chinese medicine) per patient for those receiving Chinese medicine therapy were 18,798 RMB (approximately NT$ 82,711) (Nie et al., 2023). This study included a total of 43,122 NSCLC patients from 2007 to 2018. The 5-year average total medical expenses per person for those receiving adjunctive TCM therapy were NT$ 1,385,021, equivalent to an average annual total medical expense of NT$ 277,004. Although there are numerous studies exploring medical costs and cost-effectiveness, many of them focus on diseases other than lung cancer. Some studies have also investigated the cost-effectiveness of Chinese medicine therapy for non-cancerous conditions (Li et al., 2014; Xu et al., 2020). Comparing different diseases is challenging due to variations in healthcare capabilities, insurance reimbursement systems, and economic environments among different countries, making direct comparisons and inferences difficult.

In this study, taking total cost as an example, the tracked calculation showed that the 5-year average total medical expenses per person for those with adjunctive TCM therapy (NT$ 1,385,021) were higher than those without such therapy (NT$ 1,236,988). Additionally, the average survival years for those with adjunctive TCM therapy (2.931 years) were longer than those without adjunctive TCM therapy (2.763 years). The CER for different cancer stages, regardless of whether there was adjunctive TCM therapy, increased with higher cancer stages. For the group with adjunctive TCM therapy, the ICER for an additional year of survival was NT$ 880,908, which is 1.47 times Taiwan’s *per capita* GDP. The stage-specific ICER was highest for stage II patients at 994,722 NT$/year, equivalent to 1.67 times Taiwan’s *per capita* GDP. All ICER values were below the WHO’s suggested threshold of three times the GDP, indicating cost-effectiveness. It is noteworthy that stage I NSCLC patients did not show an increase in survival after the intervention of adjunctive TCM therapy, indicating a lack of cost-effectiveness in this subgroup.

Furthermore, utilizing bootstrapping simulation for sensitivity analysis, the cost-effectiveness of adjunctive TCM therapy was found to be 100% in line with the economic benefits of WTP within 3 times the GDP. Despite the slightly higher medical costs associated with adjunctive TCM therapy compared to no such therapy, it is highly recommended for NSCLC patients, especially for those in stages III and IV, where the cost-effectiveness is even more pronounced. Adjunctive TCM therapy not only extends the survival time of patients but also reduces complications associated with lung cancer and treatment-related discomfort (Lam et al., 2017; Cai et al., 2018; Kuo et al., 2018; Lee et al., 2018).

This study’s results regarding commonly used single herbs and formulas in adjunctive therapy for NSCLC patients differ slightly from previous studies. A 2017 study in Taiwan found that the most frequently used formula was Qing Zao Jiu Fei Tang (Liao et al., 2017), whereas in our study, it was San Jhong Kuei Jian Tang. This discrepancy might be due to the different study periods; the previous study used NHIRD from 2000 to 2009, while our study covers 2007–2013, with little overlap in the years. San Jhong Kuei Jian Tang is effective in clearing heat, detoxifying, dispersing masses, and reducing swelling. It can be used to treat scrofula, lymphadenitis, acute folliculitis, thyroiditis, and goiter (Chinese Medicine formulae from the, 2024). On the other hand, Qing Zao Jiu Fei Tang is known for its ability to clear dryness and moisten the lungs, and it is suitable for treating pneumonia, pulmonary tuberculosis, bronchial asthma, acute and chronic bronchitis, emphysema, lung cancer, urticaria, and laryngitis (DOCMAP, 2024). Therefore, Qing Zao Jiu Fei Tang acts as an anti-cancer formula, while San Jhong Kuei Jian Tang primarily treats NSCLC complications. A 2020 Taiwanese study also found that Scleromitrion diffusum was the most commonly used single herb (Yeh et al., 2020), consistent with our study results. Scleromitrion diffusum is known for clearing heat, detoxifying, promoting diuresis, and alleviating stranguria. It has antibacterial, anti-inflammatory, and anti-tumor properties; it also inhibits spermatogenesis while protecting the liver and promoting bile secretion (KMWEB, 2024).

In this study, the ten most commonly used single herbs and formulas for NSCLC patients receiving adjunctive TCM therapy include the single herbs Scleromitrion diffusum, Platycodon grandiflorus, Astragalus mongholicus, Fritillaria thunbergii, Houttuynia cordata, Scutellaria barbata, Scutellaria baicalensis, Prunus mandshurica, Rheum rhabarbarum, and Taraxacum mongolicum. These single herbs are all related to the treatment of NSCLC. The formulas related to anti-cancer therapy include Siang Sha Liou Jyun Zih Tang, Bai He Gu Jin Tang, Qing Zao Jiu Fei Tang, Bu Zhong Yi Qi Tang, and Ganlu Yin. Other formulas, such as San Jhong Kuei Jian Tang, Xiao Chai Hu Tang, Jiawei Siaoyao San, Bansia Siexin Tang, and Wen Dan Tang, have anti-inflammatory properties and enhance the immune system, as well as strengthening the digestive system. These can help alleviate the complications of NSCLC and mitigate the side effects of Western medications (Su et al., 2020; Lo and Hung, 2021; Zhang et al., 2021).

## Strengths

This study possesses several strengths: Firstly, it utilizes nationwide statistical data, collecting information on NSCLC patients over a 7-year period (2007–2013) and tracking their outcomes for 5 years. Secondly, the study conducts survival and cost-effectiveness analyses for NSCLC, considering both undifferentiated by cancer stage and segmented by various cancer stages. Thirdly, the survival analysis in this study addresses and excludes immortal time bias, ensuring the accuracy of the results. Additionally, the CEA incorporates a 3% discount and CPI correction. Finally, the study employs PSM at a 1:5 ratio to control for variables such as cancer stage, gender, age, monthly salary, education level, marital status, CCI, and medical history (diabetes, liver cirrhosis, renal failure, cerebrovascular disease, and chronic obstructive pulmonary disease), as well as whether surgery was performed within 90 days. This approach ensures precise matching between NSCLC patients who received adjunctive TCM therapy and those who did not, accounting for various relevant factors.

## Limitations

This study has some limitations. In the treatment of NSCLC, it excludes the increasingly popular immunotherapy, as literature indicates a significant benefit to survival. However, since this study extracted cancer data up to 2013 and observed until 2018, excluding studies on immunotherapy might not reflect the current overall survival outcomes. The study’s sample comes from the NHIRD, and it ignores real-world patient compliance with TCM and Western medicine when conducting analyses based on diagnostic and procedure codes. Unmeasured confounders, or unquantifiable interfering factors, may exist in retrospective studies and potentially influence the analysis results. Smoking status is an important factor when assessing the mortality risk in NSCLC patients; however, there is no record of smoking status in the NHIRD. Additionally, data on a small portion of out-of-pocket TCM services (such as herbal decoctions) cannot be obtained from the NHIRD. If out-of-pocket TCM services could enhance efficacy, this aspect is not included in the comparative analysis.

## Conclusion

For NSCLC patients, the use of adjunctive TCM therapy for 181–365 days significantly reduces the risk of mortality by 12%, especially in stage IV NSCLC patients, where there is a significant 14% reduction in the risk of mortality. Whether considering primary diagnosis costs, primary and secondary diagnosis costs, or total costs, the CER for patients with adjunctive TCM therapy is higher than those without. The ICER for adjunctive TCM therapy in NSCLC stages II to IV is 994,722 NT$/year, 568,869 NT$/year, and 724,791 NT$/year, respectively, all within the economic benefit of WTP prices within 3 times the *per capita* GDP. In summary, adjunctive TCM therapy for NSCLC patients not only reduces the mortality rate but also demonstrates cost-effectiveness. Scleromitrion diffusum and San Jhong Kuei Jian Tang are the most commonly prescribed single herbs and formulas, respectively.

## Data Availability

The original contributions presented in the study are included in the article/supplementary material, further inquiries can be directed to the corresponding author.

## Abbreviations

NSCLC, Non-Small Cell Lung Cancer; NHIRD, National Health Insurance Research Database; TCM, Traditional Chinese medicine; ICD, International Classification of Diseases; CPI, Consumer Price Index; CEA, cost-effectiveness analysis; ICER, incremental cost-effectiveness ratio; CCI, Charlson Comorbidity Index; HR, Hazard ratio; CI, confidence interval; NT$, New Taiwan dollar; WTP, willingness to pay; GDP, Gross domestic product.
